# A Minireview of Microfluidic Scaffold Materials in Tissue Engineering

**DOI:** 10.3389/fmolb.2021.783268

**Published:** 2022-01-11

**Authors:** Anh Tong, Roman Voronov

**Affiliations:** ^1^ Otto H. York Department of Chemical and Materials Engineering, Newark College of Engineering, New Jersey Institute of Technology, Newark, NJ, United States; ^2^ Department of Biomedical Engineering, Newark College of Engineering, New Jersey Institute of Technology, Newark, NJ, United States

**Keywords:** microfluidic scaffold, biomaterial, tissue engineering, regenerative medicine, biomanufacturing, 3D printing

## Abstract

In 2020, nearly 107,000 people in the U.S needed a lifesaving organ transplant, but due to a limited number of donors, only ∼35% of them have actually received it. Thus, successful bio-manufacturing of artificial tissues and organs is central to satisfying the ever-growing demand for transplants. However, despite decades of tremendous investments in regenerative medicine research and development conventional scaffold technologies have failed to yield viable tissues and organs. Luckily, *microfluidic* scaffolds hold the promise of overcoming the major challenges associated with generating complex 3D cultures: 1) cell death due to poor metabolite distribution/clearing of waste in thick cultures; 2) sacrificial analysis due to inability to sample the culture non-invasively; 3) product variability due to lack of control over the cell action post-seeding, and 4) adoption barriers associated with having to learn a different culturing protocol for each new product. Namely, their active pore networks provide the ability to perform automated fluid and cell manipulations (e.g., seeding, feeding, probing, clearing waste, delivering drugs, etc.) at targeted locations *in-situ*. However, challenges remain in developing a biomaterial that would have the appropriate characteristics for such scaffolds. Specifically, it should ideally be: 1) *biocompatible*—to support cell attachment and growth, 2) *biodegradable*—to give way to newly formed tissue, 3) *flexible*—to create microfluidic valves, 4) *photo-crosslinkable*—to manufacture using light-based 3D printing and 5) *transparent*—for optical microscopy validation. To that end, this minireview summarizes the latest progress of the biomaterial design, and of the corresponding fabrication method development, for making the microfluidic scaffolds.

## Introduction

According to the U.S. Department of Health & Human Services, nearly 107,000 people in the U.S needed a lifesaving transplant in 2020, while only ∼35% of them have received it. Yet, almost no FDA-approved (Administration, U.S.F.a.D., 2019) artificial organs are commercially available today—3 decades after the inception (Langer and Vacanti, 1993) of tissue engineering and after billions of dollars invested into its development. Therefore, a new approach to biomanufacturing is needed. Yet, there are still major obstacles restricting the progress of 3D culturing technologies towards the biomanufacturing of complex organs and tissue recreation *in vitro* (Ye et al., 2018):

1) Product Size Limitations—due to the lack of an active vasculature and blood circulation within lab-grown tissues, it is difficult to deliver nutrients to/clear metabolic waste from the inner pore spaces of organ-sized scaffolds. As a result, cell survival in the deep portions of the large scaffolds is compromised due to hypoxia, metabolic waste accumulation and insufficient nutrient availability.
2) Sacrificial Analysis—due to the inability to sample cells and fluids from within scaffolds nondestructively, and because live long-term 3D microscopy is challenging. This makes it necessary to perform destructive testing at the conclusion of each experiment, such as histological sectioning or crushing the scaffold for plate reader assays. As a result, a different sample must be cultured for each new time point. This balloons the cost of experiments and slows down the scientific progress tremendously.
3) Product Variability—due to the absence of a native supervision over the *in vitro* cell behavior, and lack of access to the cells post seeding, there is no orchestration over their actions within the scaffolds. For example, even if one were to bioprint the perfect artificial tissue (i.e., deposit the cells into precise locations within a scaffold), the cells within it would be free to do a number of undesirable things afterwards: a) migrate away uncontrollably (Wu et al., 2014), b) differentiate into the wrong tissue type (e.g., a patient grew mucus tissue in her spine, as a result of stem cell therapy) (Dlouhy et al., 2014), and c) deposit tissue in the wrong locations and occlude the scaffold pores. All this leads to nonviable tissue and poor product consistency.
4) Technology Adoption Barriers—Even if one could generate the perfect tissue in a laboratory, training hospital staff in custom culturing protocols for each new product remains another critical hurdle holding back biomanufacturing technologies from entering the market. Therefore, there is a need for an automated 3D cell culturing platform capable of minimizing the variations between experiments.

**FIGURE 1:**
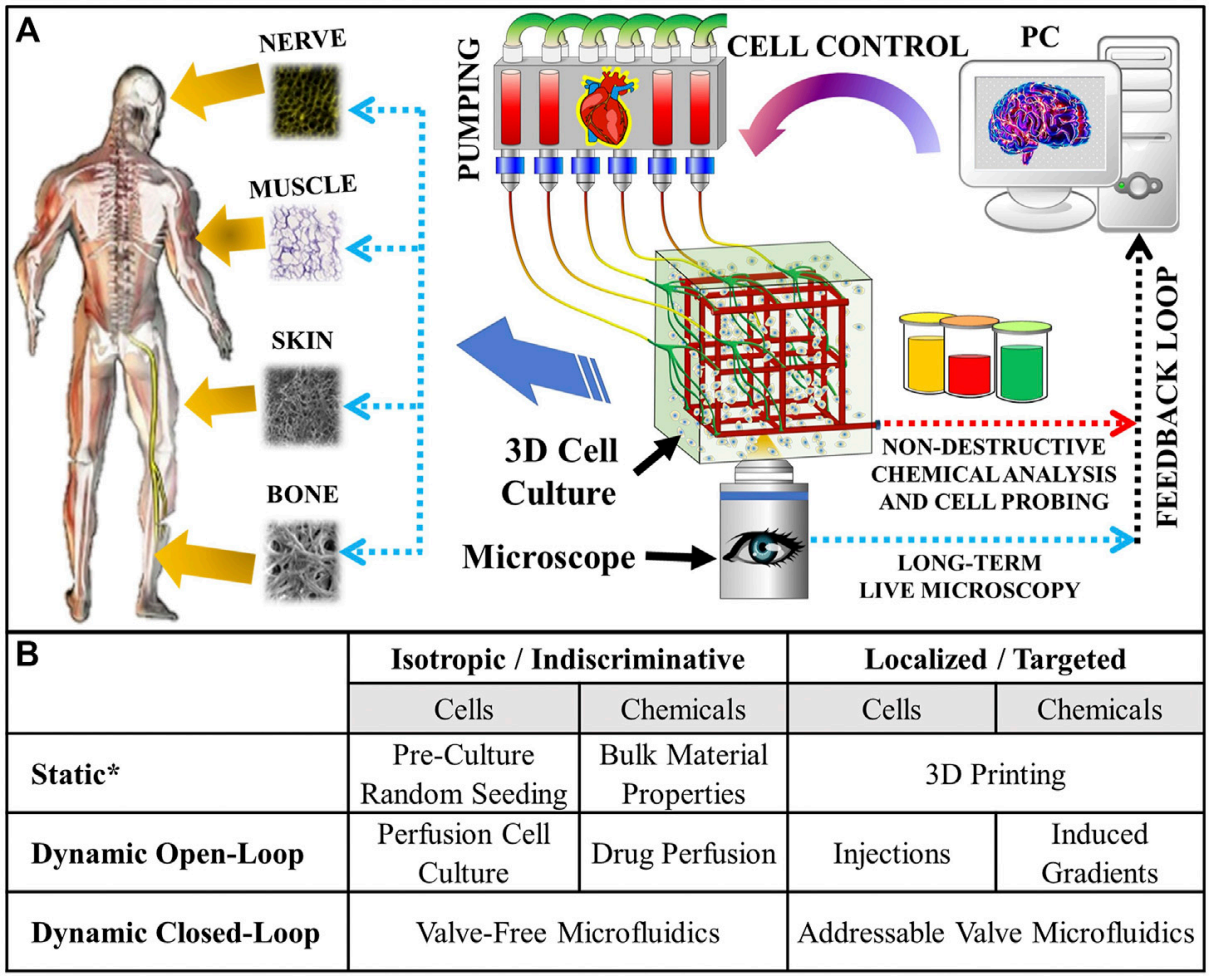
**(A)** Concept of the microfluidic scaffold with active “vasculature” that enables targeted real-time fluid and cell manipulation within the cultured tissue. External pumping acts like a “heart”, while the whole process is orchestrated by a computer acting like a “brain”. The computer’s closed-loop responses are based on feedback from non-destructive chemical analysis and long-term live microscopy throughout the entire duration of the culture. Viable artificial tissue is cultured automatically and reproducibly. **(B)** Types of cell and/or fluid manipulations possible in tissue engineering scaffolds. *“Static” refers to one-time manipulations—usually performed at the start of the experiment. The addressable valve microfluidics hold numerous advantages over the conventional culturing methods, including the potential for enabling minimally disruptive localized additive (e.g., cell and drug delivery) and subtractive (e.g., probing cell secretions, collecting biopsies, making corrections by removing tissue overgrowth, etc.) manipulations, which can be used to implement *closed* (i.e., in response to real time feedback) loop controls. These are essential for growing complex spatial tissue patterns that evolve over time: for example, some bones in our bodies start out as cartilage and only subsequently calcify through endochondral ossification(Scotti et al., 2010; Farrell et al., 2011).

Recently, a lot of progress has been made in generating *microscopic* 3D cell culturing environments (e.g., posts, fibers, etc.) in microfluidics devices (Raman et al., 2013; Morrow et al., 2019). Therefore, adopting the *microscopic* cell manipulation technologies for the creation of *macroscopic* cultures (aka “microfluidic scaffolds”) can potentially overcome *all* of the bottlenecks above by providing the following key elements: 1) An Active “Vasculature” for distributing metabolites and clearing waste throughout organ-sized scaffolds; 2) Non-Disruptive Mini-Probing of the cells and of the fluids within them for *ex-situ* analysis [as was done in Supplementary Figure S1 of (Tong et al., 2020)]; 3) Long-term Live Microscopy validation of the cell/fluid probing/assaying in #2, where possible (e.g., at objective focal length depths within the scaffold, assuming an optically transparent scaffold material); 4) Tissue Modulation via cell and bio-active chemical (e.g., chemo-attractants, growth and differentiation factors, drugs, etc.) delivery, in order to minimize product variability; and 5) Automated Spatiotemporal Control over the tissue development in a closed-loop manner, based on optical and chemical assaying feedback, in order to enable computer-driven culturing. The overall idea is depicted in Figure 1A.

Therefore, the microfluidic scaffolds offer numerous advantages over the conventional culturing/biofabrication methods (see Figure 1B). And, this technology has been applied to create numerous tissue type and disease models, as well as biosensors: kidney (Miller et al., 2012), heart (Zhang et al., 2018), lung (Grigoryan et al., 2019), liver (Zhang et al., 2018; Grigoryan et al., 2019), blood coagulation (Zhang et al., 2016), vascularization (King et al., 2004; Wang et al., 2010; Shen et al., 2019), cancer (Bettinger et al., 2006; Pimentel et al., 2018), and implantable biosensors (Zhao et al., 2016). However, there are two main challenges preventing from unlocking the full potential of these promising technologies:

1) An automated plumbing architecture, which offers numerous advantages over valve-free microfluidic scaffolds and other conventional biomanufacturing/culturing methods (see Figure 1B), capable of localized fluid and/or cell manipulations needs to be developed: Although several attempts have been made in the past to create the microfluidic scaffolds (King et al., 2004; Bettinger et al., 2006; Bettinger et al., 2007; Wang et al., 2010; Miller et al., 2012; Zhang et al., 2016; Zhao et al., 2016; Pimentel et al., 2018; Zhang et al., 2018; Grigoryan et al., 2019; Shen et al., 2019), these studies mostly focused on developing novel biodegradable *materials* for this purpose, while the plumbing in these devices consisted of rudimentary straight-through pores (i.e., without any valves or automation) for simplicity. Most recently, however, our group has laid a foundation for an automated “addressable” plumbing that uses Polydimethylsiloxane (PDMS) microfluidic *valves* for achieving 2D (with the 3D version being in progress) fluid and/or cell manipulations at targeted XY locations within a single culturing space (Tong et al., 2020). A big advantage of this technology is that it is scalable to *organ-sized* scaffolds and can enable single cell manipulation (which is ideally required for complex tissue patterning and nondisruptive analysis *in-situ*); and,
2) The scaffold’s material should be *biocompatible* (i.e., nontoxic and be able to support cell adhesion) and *biodegradable* (with a tunable degradation time). This is to give way to the newly formed tissue synthesized by the cells in the 3D culture. Ideally, the degradation rate of a scaffold should be synchronized with the *in vivo* tissue regeneration rate: for example, soft tissues (e.g., skin) require around 14–21 days, while hard tissues (e.g., bone) need 8 to 12 + weeks for complete healing (Yildirimer et al., 2012; Sukpaita et al., 2019; Choi et al., 2021; Ma et al., 2021). Therefore, ∼6 weeks is an optimal *half-life* requirement for a material to be considered to have a degradation long enough to be used for long-term tissue regeneration strategies. Furthermore, the material should have mechanical properties similar to that of PDMS (i.e., an elastic modulus of 1.32–2.97 MPa and an elongation at break (i.e., stretchability) of 40% (Johnston et al., 2014)), in order to make the microfluidic valves (as in the addressable plumbing published by our group (Tong et al., 2020)) needed for the localized fluid and cell manipulations within the scaffold. Moreover, the material should also be *photo-crosslinkable,* if a light-based fabrication approach (e.g., stereolithography 3D printing) would be used to manufacture the microfluidic scaffolds. Although, methods that don’t require crosslinking (e.g., micro-extrusion 3D printing) could be used instead, the light-based ones tend to yield the highest resolution (which is desired for the microfluidic scaffolds capable of precise cell and fluid manipulation *in situ* (Tong et al., 2021)). Lastly, another consideration is the *refractive index (RI)* of the material, which for optical microscopy should ideally be in between that of water = 1.33 and of glass = 1.52 (Bashkatov and Genina, 2003; Ürek et al., 2021). Although not a hard requirement, it would reduce the light aberrations experienced by water-dipping objectives commonly used by the popular 3D long term fluorescence imaging methods (e.g., Lattice Light Sheet Microscopy).

Hence, given that the foundation for the addressable plumbing and the automation design have been described in our recent publication (Tong et al., 2020), the remainder of this minireview is dedicated to the development of the novel *materials* for the microfluidic scaffolds. Although many excellent reviews of materials used for microfluidics *devices* have been published in the past (Huang et al., 2011; Papaefthymiou, 2013; Ren et al., 2013; Hou et al., 2017; Mou and Jiang, 2017; Convery and Gadegaard, 2019; Xie et al., 2020), none of them have focused on the creation of microfluidic *scaffolds* with an addressable (i.e., with valves) plumbing. Thus, the discussion is focused solely on the materials needed for creating the envisioned device. Furthermore, it does so without assuming a tissue type that the scaffolds would be used to culture. Hence, the reviewed material properties do not account for tissue-specific requirements, such as cell adherence and mechanotransduction. Instead, it is presumed that such customizations would be accomplished by coating the interior pore space of the envisioned device with the tissue-specific proteins *via* its microfluidic plumbing.

## Microfluidic Scaffold—Materials and Manufacturing

According to our literature search, the different types of biomaterials, and corresponding fabrication techniques, developed for the construction of the microfluidic scaffolds are summarized in Table 1.

**TABLE 1:** Comparison of candidate biomaterials that have been used, or could potentially be used, to make microfluidic scaffolds.

Material	Fabrication Type	Automated multilayer alignment	Photo- crosslinkable	Optically Transparent	Slow degradation	Stretchable	Flexible
*Naturally-derived Materials*							
Fibrin	3D	✓	X	X	X	✓	X
Alginate	3D	✓	X	✓	X	✓	X
Matrigel	3D	✓	X	✓	X	N/A	X
Silk Fibroin	2D	X	X	✓	✓	X	✓
Gelatin	3D	✓	X	✓	X	X	X
GelMA	3D	✓	✓	✓	X	✓	X
*Synthetic Materials*							
PLGA	2D	X	X	✓	✓	X	✓
PGS	2D	X	X	N/A	X	✓	✓
APS	2D	X	X	✓	✓	✓	✓
POMaC	3D	✓	✓	N/A	✓	✓	✓
F127-DA	2D	X	✓	✓	X	✓	X
PEGDA	3D	✓	✓	✓	X	✓	✓

For reference, we consider a material to be: “Optically Transpartent”, if its RI is between that of water (1.33) and of glass (1.52) (Bashkatov and Genina, 2003; Ürek et al., 2021); “Flexible”, if its elastic modulus is within the PDMS range of 1.32–2.97 MPa (Johnston et al., 2014); “Stretchable”, if its “Elongation at Break” is greater than that of PDMS (which corresponds to a value of 40%) (Johnston et al., 2014). Lastly, if a material has a degradation half-life of at least 6 weeks, it is considered to be able to support long-term tissue growth.

### Naturally Derived Materials

The first line of materials reviewed here belongs to those derived from *natural* sources, such as: Alginate from seaweed, Gelatin from animal skin, Silk from worm cocoons, Matrigel from extracellular matrix and Fibrin from blood. These are attractive for scaffold manufacturing due to their biocompatibility with cell and tissue growth.

**Extracellular Matrix (ECM) and Cell Wall Materials (e.g., Fibrin, Matrigel, and Alginate)** (Miller et al., 2012): 3D perfusable vascular networks were created by casting of Fibrin, Alginate, or Matrigel hydrogel solutions onto a dissolvable carbohydrate-glass lattice via a 3D printed sacrificial molding technique. All three gels were polymerized either chemically or thermally (as opposed to via photo-crosslinking). Once a hydrogel was solidified, the sacrificial lattice was dissolved out, generating a complex 3D vascular network without the need for any manual alignment or bonding. The optical properties of Alginate and Matrigel, with an RI ∼1.335–1.34.(Choi et al., 2015; Funane et al., 2018), are close to that of water; while the Fibrin’s RI of 1.53–1.62 (Kirichenko et al., 2021) is slightly higher than that of glass. Despite these attractive properties, however, these hydrogels are too soft for microfluidic valve fabrication: the elastic modulus of Alginate is ∼0.1–10 KPa (Candiello et al., 2013), of Matrigel ∼0.14–1.3 KPa (Kiss et al., 2011) and of Fibrin ∼9.29–30 kPa (Murphy and Leach, 2012; Bachmann et al., 2020). Thus, despite having an excellent elongation at break (e.g., ∼400% for Alginate and 52% for Fibrin (Underwood et al., 2001)), the mechanical properties of these hydrogels are significantly weaker than that of the PDMS (increasing the likelihood of device collapse and deformation). Furthermore, they have shown to degrade too rapidly *in vivo*: ∼1 week (Benavides et al., 2015; Shkand et al., 2016; Trujillo et al., 2019).

**Silk Fibroin** (Zhao et al., 2016): A microfluidic Silk Fibroin hydrogel scaffold has been created via soft lithography using PDMS stamps and sacrificial molding. To the best of our knowledge, this is the only transparent (RI = 1.34 (Shan et al., 2018)) biodegradable material in Table 1 that has actually been used to create functional microfluidic valves. The Silk Fibroin was crosslinked via enzymatic reactions. Next, new layers were manually cast onto a semi-crosslinked ones to achieve good bonding. It has an excellent tunable degradation half-life from hours to years. However, it has not been shown that the material can be used to generate valve sizes for single cell manipulation—the construct’s feature sizes in (Zhao et al., 2016) were quite large: 800–1,200 μm, with a layer thickness of ∼2,500 µm. Therefore, the fabrication method needs to be optimized to achieve a finer feature resolution of ∼100 µm for precise cell and fluid manipulations within the microfluidic scaffolds. Another drawback of Silk Fibroin is that its elastic modulus of 1 kPa - 1 MPa (Zhao et al., 2016) and elongation at break of 15–35% (Gu et al., 2020) are lower than that of the PDMS. In fact, a PDMS support was used with the device in (Zhao et al., 2016).

**Gelatin** (Pimentel et al., 2018)**/Gelatin Methacrylate (GelMA)** (Zhang et al., 2016): 3D perfusable vascular networks were fabricated from Gelatin/GelMA by casting an aqueous hydrogel solution on a 3D printed sacrificial molding. The mold was then dissolved within the solidified hydrogel to generate complex 3D vascular networks without any manual alignment or bonding. Both of the materials are transparent, with optical properties close to that of water (RI = 1.35–1.39 (Fu et al., 2017; Rinawati et al., 2018)). After being crosslinked chemically, Gelatin had an elastic modulus of ∼0.3–20 kPa (Zhao et al., 2016) and an elongation at break of ∼70% (Lee et al., 2019). GelMA, on the other hand, is a UV crosslinkable material. Its elastic modulus is around 3.08–184.52 KPa (Wu et al., 2019), and its elongation at break can be as large as 80% (Shin et al., 2012). Unfortunately, both Gelatin and GelMA have a really short degradation half-life: ∼25 days for gelatin (Saito and Tabata, 2012) and ∼3 h for GelMA (Zhu et al., 2019). Finally, like the other *naturally* derived hydrogels, Gelatin/GelMA are generally so soft that they require other materials to serve as mechanical support(Huang et al., 2011).

### Synthetic Materials

The second group of materials reviewed here are *artificially* designed to have better mechanical properties than their *naturally* derived counterparts. Specifically, most of the latter materials are too soft to make microfluidic valves. Hence the *synthetic* materials have been made to improve stiffness and stretchability, and are therefore, generally better candidates for the microfluidic scaffolds.

**Poly-lactic-co-glycolic acid (PLGA)** (King et al., 2004): Patterned 2D PLGA layers were with complex branching pore networks has been created via soft lithography. Once microstructured the films were fabricated, manual layer-by-layer alignment and thermal bonding were used to stack them into a 3D scaffold. Thus, given the use of manual fabrication, we are not aware of this material being compatible (i.e., photo-crosslinkable) with SLA 3D printing. Besides that, PLGA has an elastic modulus of 1.4–2.8 MPa (Barrett and Yousaf, 2009), which makes sufficiently rigid (like PDMS). Furthermore, its RI of 1.47 (Shan et al., 2018) is close to the range of water and glass (1.33–1.52), making it suitable to be used with optical microscopy. Moreover, PLGA has an attractive degradation half-life of 5–6 weeks (Gentile et al., 2014). However, its maximum elongation at break is just 10%, making PLGA making it likely that the microfluidic valves made from this material could tear during use.

**Poly(glycerol sebacate) (PGS)** (Bettinger et al., 2006): Microfluidic scaffolds made out of up to five layers of PGS have been created using soft lithography with manual alignment and thermal bonding. The material is reported to be transparent, though we were unable to find out its exact RI. It also has a good elastic modulus of 0.77–1.9 MPa (Vogt et al., 2021) and a high elongation at break 267% (Barrett and Yousaf, 2009). However, it is known to degrade too fast (a half-life of ∼21 days (Pomerantseva et al., 2009)) for long-term tissue regeneration. Therefore, more work needs to be done to slow down its degradation time and to make it crosslinkable for light-based fabrication.

**Poly(1,3-diamino-2-hydroxypropane-co-polyol sebacate) (APS)** (Wang et al., 2010): Single layer APS vascular networks were created via soft lithography in conjunction with thermal curing and bonding. The material is transparent with an RI = 1.5 (Thomas et al., 2012) and has a degradation half-life that is tunable between 6 and 100 weeks (Bettinger et al., 2009). Additionally, with an elastic modulus of 0.56–4.34 MPa (Bettinger et al., 2009; Wang et al., 2010) and an elongation at break of 21–151% (Bettinger et al., 2009), APS’ mechanical properties can be made sufficiently close to that of the PDMS. However, given that it has not been made photo-crosslinkable and has only been used with 2D manufacturing methods, better approaches need to be developed for fabricating 3D microfluidic scaffolds with valves out of the APS.

**Poly(octamethylene maleate (anhydride) citrate) (POMaC)** (Zhang et al., 2018): A multilayer POMaC scaffold with a branching microchannel network was created via 2D soft lithography, in conjunction with ultraviolet light (UV) photo-crosslinking, manual alignment and inter-layer bonding using UV irradiation. Like PGS, the POMaC scaffold was shown to be transparent, though its RI has not been measured. Additionally, the material has excellent mechanical properties: an elastic modulus of 0.03–1.54 MPa and an elongation at break of 48–534% (Tran et al., 2010). Consequently, 25–50 µm thin vascular walls of the POMaC scaffold proved to be elastic enough to support pulsating blood flow in an artificial cardiac tissue implanted into a rat. Furthermore, in a different application, Digital Micromirror Device Projection 3D printing was used to photo-crosslink the material(Cha et al., 2014). Hence, POMaC is one of the best candidate materials that has all (less the missing RI) of the desired properties in Table 1.

**Pluronic F127 di-acrylate (F127-DA)** (Shen et al., 2019): A single layer perfusable microchannel network was created from the F127-DA hydrogel via a serial soft lithography using 3D printed molds and PDMS stamps. The material was photo-crosslinked under UV irradiation. It is transparent and is non-swelling at 37°C, which helps the microfluidic construct to maintain its desired channel morphology. Furthermore, it can be stretched to an incredible 11x (or 1,100%) of its original length before breaking. Additionally, the F127 hydrogel is transparent with an RI lower than 1.4 (Galy et al., 2020). However, its elastic modulus of just ∼75 kPa, making failure more likely due to the mechanical properties of this material significantly weaker than that of the PDMS. For example, the microfluidic construct made from this material has been shown to have a burst limit at ∼600 mmHg (11 psi) due to leakage from its tubing connections. Hence, the F127-DA is potentially unsuitable for microfluidic valve fabrication, since a bursting limit pressure of ∼2x (i.e., 20–25 psi) is needed to maintain microfluidic valve integrity during operation(Kellogg et al., 2014; Tong et al., 2020). Furthermore, F127-DA has a relatively short degradation half-life of ∼21 days. Therefore, more work would need to be done on improving its mechanical and degradation properties, before the material can be used for 3D microfluidic scaffolds with valves.

**Photocrosslinkable poly(ethylene glycol) diacrylate (PEGDA)** (Grigoryan et al., 2019): Multiple 3D perfusable multi-vasculature networks were created out of PEGDA using rapid prototyping SLA 3D printing. The construct was crosslinked automatically via UV exposure, and no manual bonding was needed. Similar to POMaC, PEGDA is a transparent material with an RI = 1.35 (Choi et al., 2013) which is close to that of water. Furthermore, it can be synthesized to exhibit a wide range of mechanical flexibility values varying from relatively soft ∼0.1 MPa to very stiff ∼18 MPa (Skornia et al., 2007; Chen et al., 2018). And PEGDA has an elongation at break ranging from ∼21 to above 65% (Chen et al., 2018; Feng et al., 2020). It’s one downside, however, is a short degradation half-life of just ∼25 days. Nonetheless, with future work, it can likely get tuned to the appropriate levels. Therefore, PEGDA is also considered to be a potential candidate for the microfluidic scaffolds, which meets most of the criteria outlined in Table 1.

## Conclusion

In conclusion, microfluidic scaffolds hold the potential to revolutionize the biomanufacturing of artificial tissues and organs. However, an easily manufacturable biomaterial capable of supporting cell growth for extended periods of time, while maintaining the flexible microfluidic valves and optically transparent properties, needs to be found to make this happen. We have reviewed the potential candidate materials available in the literature and have determined that most *naturally* derived hydrogels (e.g., Gelatin/GelMA, and ECM/Cell Wall hydrogels) generally lack the mechanical properties for generating sturdy microfluidic valves. For example, Silk fibroin had to be used in conjunction with supports made from other (stronger) materials, like PDMS and Acrylic. They also tend to be leaky, which makes the hydrogels unlikely candidates for fabrication of the 3D microfluidic scaffolds with non-rudimentary plumbing. Consequently, synthetic materials (e.g., PLGA, PGS, APS, POMaC, F127-DA, and PEGDA) have been specifically designed to have the mechanical properties that are more tailored towards the microfluidic valve fabrication, while still retaining the biocompatibility and degradation profiles of an implantable material. Among these, the best overall candidate materials for making the microfluidic plumbing with valves appear to be POMaC and PEGDA, because they fit most of the criteria in Table 1. While the former appears to meet all the requirements, we were not able to find data on its transparency. Meanwhile, the latter has the drawback of having a relatively short degradation half-life of ∼25 days. However, even with this duration, the material can still be used for tissues that do not require long-term mechanical support. Ideally, though, we anticipate that a more stable version will soon be designed to accommodate all tissue types. Lastly, despite some of the materials being photocrosslinkable, all the microfluidic scaffolds reviewed in this manuscript were made either via solvent casting or manual stacking/bonding of 2D layers fabricated via soft lithography. Thus, the manufacturing approaches need to be improved also, ideally to use automated high-resolution approaches, such as stereolithography 3D printing. Once such a material is designed, the advanced microfluidic scaffolds will, without a doubt, begin to play a more dominant role in tissue engineering and biomanufacturing.

## References

[B1] Administration, U.S.F.a.D. (2019). Approved Cellular and Gene Therapy Products. [Online]. U.S. Food and Drug Administration. Available: https://www.fda.gov/vaccines-blood-biologics/cellular-gene-therapy-products/approved-cellular-and-gene-therapy-products (Accessed 0617, 2019).

[B2] Bachmann B. Spitz S. Schädl B. Teuschl A. H. Redl H. Nürnberger S. (2020). Stiffness Matters: fine-tuned Hydrogel Elasticity Alters Chondrogenic Redifferentiation. Front. Bioeng. Biotechnol. 8, 373. 10.3389/fbioe.2020.00373 32426347 PMC7204401

[B3] Barrett D. Yousaf M. (2009). Design and Applications of Biodegradable Polyester Tissue Scaffolds Based on Endogenous Monomers Found in Human Metabolism. Molecules 14, 4022–4050. 10.3390/molecules14104022 19924045 PMC6255442

[B4] Bashkatov A. N. Genina E. A. (2003). “Water Refractive index in Dependence on Temperature and Wavelength: a Simple Approximation,” in Saratov Fall Meeting 2002: Optical Technologies in Biophysics and Medicine IV, Saratov, Russia, 1-4 October 2002 (Bellingham, WA: International Society for Optics and Photonics), 393–396.

[B5] Benavides O. M. Quinn J. P. Pok S. Petsche Connell J. Ruano R. Jacot J. G. (2015). Capillary-Like Network Formation by Human Amniotic Fluid-Derived Stem Cells within Fibrin/Poly(Ethylene Glycol) Hydrogels. Tissue Eng. A 21, 1185–1194. 10.1089/ten.tea.2014.0288 PMC4394886 25517426

[B6] Bettinger C. J. Bruggeman J. P. Borenstein J. T. Langer R. (2009). *In vitro* and *in vivo* degradation of poly(1,3-diamino-2-hydroxypropane-co-polyol sebacate) elastomers. J. Biomed. Mater. Res. 91 (4), 1077–1088. 10.1002/jbm.a.32306 19107786

[B7] Bettinger C. J. Cyr K. M. Matsumoto A. Langer R. Borenstein J. T. Kaplan D. L. (2007). Silk Fibroin Microfluidic Devices. Adv. Mater. 19, 2847–2850. 10.1002/adma.200602487 19424448 PMC2677821

[B8] Bettinger C. J. Weinberg E. J. Kulig K. M. Vacanti J. P. Wang Y. Borenstein J. T. (2006). Three-Dimensional Microfluidic Tissue-Engineering Scaffolds Using a Flexible Biodegradable Polymer. Adv. Mater. 18, 165–169. 10.1002/adma.200500438 PMC2744127 19759845

[B9] Candiello J. Singh S. S. Task K. Kumta P. N. Banerjee I. (2013). Early Differentiation Patterning of Mouse Embryonic Stem Cells in Response to Variations in Alginate Substrate Stiffness. J. Biol. Eng. 7, 9–14. 10.1186/1754-1611-7-9 23570553 PMC3643844

[B10] Cha C. Soman P. Zhu W. Nikkhah M. Camci-Unal G. Chen S. (2014). Structural Reinforcement of Cell-Laden Hydrogels with Microfabricated Three Dimensional Scaffolds. Biomater. Sci. 2, 703–709. 10.1039/c3bm60210a 24778793 PMC4000042

[B11] Chen J.-Y. Hwang J. Ao-Ieong W.-S. Lin Y.-C. Hsieh Y.-K. Cheng Y.-L. (2018). Study of Physical and Degradation Properties of 3D-Printed Biodegradable, Photocurable Copolymers, PGSA-Co-PEGDA and PGSA-Co-PCLDA. Polymers 10, 1263. 10.3390/polym10111263 PMC6401713 30961188

[B12] Choi M. Choi J. W. Kim S. Nizamoglu S. Hahn S. K. Yun S. H. (2013). Light-guiding Hydrogels for Cell-Based Sensing and Optogenetic Synthesis *in vivo* . Nat. Photon 7, 987–994. 10.1038/nphoton.2013.278 PMC4207089 25346777

[B13] Choi M. Humar M. Kim S. Yun S.-H. (2015). Step-Index Optical Fiber Made of Biocompatible Hydrogels. Adv. Mater. 27, 4081–4086. 10.1002/adma.201501603 26045317 PMC4503511

[B14] Choi W. S. Kim J. H. Ahn C. B. Lee J. H. Kim Y. J. Son K. H. (2021). Development of a Multi-Layer Skin Substitute Using Human Hair Keratinic Extract-Based Hybrid 3D Printing. Polymers 13, 2584. 10.3390/polym13162584 34451127 PMC8401121

[B15] Convery N. Gadegaard N. (2019). 30 Years of Microfluidics. Micro Nano Eng. 2, 76–91. 10.1016/j.mne.2019.01.003

[B16] Dlouhy B. J. Awe O. Rao R. C. Kirby P. A. Hitchon P. W. (2014). Autograft-derived Spinal Cord Mass Following Olfactory Mucosal Cell Transplantation in a Spinal Cord Injury Patient. J. Neurosurg. Spine. 21 (4), 618–622. 10.3171/2014.5.spine13992 25002238

[B17] Farrell E. Both S. K. Odörfer K. I. Koevoet W. Kops N. O'brien F. J. (2011). *In-vivo* Generation of Bone via Endochondral Ossification by *In-Vitro* Chondrogenic Priming of Adult Human and Rat Mesenchymal Stem Cells. BMC Musculoskelet. Disord. 12, 31. 10.1186/1471-2474-12-31 21281488 PMC3045394

[B18] Feng J. Zheng Y. Bhusari S. Villiou M. Pearson S. Campo A. (2020). Printed Degradable Optical Waveguides for Guiding Light into Tissue. Adv. Funct. Mater. 30, 2004327. 10.1002/adfm.202004327

[B19] Fu F. Chen Z. Zhao Z. Wang H. Shang L. Gu Z. (2017). Bio-inspired Self-Healing Structural Color Hydrogel. Proc. Natl. Acad. Sci. USA 114, 5900–5905. 10.1073/pnas.1703616114 28533368 PMC5468601

[B20] Funane T. Hou S. S. Zoltowska K. M. Van Veluw S. J. Berezovska O. Kumar A. T. N. (2018). Selective Plane Illumination Microscopy (SPIM) with Time-Domain Fluorescence Lifetime Imaging Microscopy (FLIM) for Volumetric Measurement of Cleared Mouse Brain Samples. Rev. Scientific Instr. 89, 053705. 10.1063/1.5018846 PMC6910582 29864842

[B21] Galy T. Marszewski M. King S. Yan Y. Tolbert S. H. Pilon L. (2020). Comparing Methods for Measuring Thickness, Refractive index, and Porosity of Mesoporous Thin Films. Microporous Mesoporous Mater. 291, 109677. 10.1016/j.micromeso.2019.109677

[B22] Gentile P. Chiono V. Carmagnola I. Hatton P. (2014). An Overview of Poly(lactic-Co-Glycolic) Acid (PLGA)-Based Biomaterials for Bone Tissue Engineering. Int. J. Mol. Sci. 15, 3640–3659. 10.3390/ijms15033640 24590126 PMC3975359

[B23] Grigoryan B. Paulsen S. J. Corbett D. C. Sazer D. W. Fortin C. L. Zaita A. J. (2019). Multivascular Networks and Functional Intravascular Topologies within Biocompatible Hydrogels. Science 364, 458–464. 10.1126/science.aav9750 31048486 PMC7769170

[B24] Gu Y. Yu L. Mou J. Wu D. Zhou P. Xu M. (2020). Mechanical Properties and Application Analysis of Spider Silk Bionic Material. e-Polymers 20, 443–457. 10.1515/epoly-2020-0049

[B25] Hou X. Zhang Y. S. Trujillo-De Santiago G. Alvarez M. M. Ribas J. Jonas S. J. (2017). Interplay between Materials and Microfluidics. Nat. Rev. Mater. 2, 1–15. 10.1038/natrevmats.2017.16 PMC11237287 38993477

[B26] Huang G. Y. Zhou L. H. Zhang Q. C. Chen Y. M. Sun W. Xu F. (2011). Microfluidic Hydrogels for Tissue Engineering. Biofabrication 3, 012001. 10.1088/1758-5082/3/1/012001 21372342

[B27] Johnston I. D. Mccluskey D. K. Tan C. K. L. Tracey M. C. (2014). Mechanical Characterization of Bulk Sylgard 184 for Microfluidics and Microengineering. J. Micromech. Microeng. 24, 035017. 10.1088/0960-1317/24/3/035017

[B28] Kellogg R. A. Gómez-Sjöberg R. Leyrat A. A. Tay S. (2014). High-throughput Microfluidic Single-Cell Analysis Pipeline for Studies of Signaling Dynamics. Nat. Protoc. 9, 1713–1726. 10.1038/nprot.2014.120 24967621

[B29] King K. R. Wang C. C. J. Kaazempur-Mofrad M. R. Vacanti J. P. Borenstein J. T. (2004). Biodegradable Microfluidics. Adv. Mater. 16, 2007–2012. 10.1002/adma.200306522

[B30] Kirichenko M. N. Shkirin A. V. Chaikov L. L. Simakin A. V. Tcherniega N. V. Gudkov S. V. (2021). Structure and Refractive index of Fibrin Protofibril Aggregates According to Laser Phase Microscopy Accompanied by DLS and AFM. Biomed. Opt. Express 12, 2938–2951. 10.1364/boe.420261 34168908 PMC8194622

[B31] Kiss R. Bock H. Pells S. Canetta E. Adya A. K. Moore A. J. (2011). Elasticity of Human Embryonic Stem Cells as Determined by Atomic Force Microscopy. J. Biomech. Eng. 133 (10), 101009. 10.1115/1.4005286 22070334

[B32] Langer R. Vacanti J. P. (1993). Tissue Engineering. Science 260, 920–926. 10.1126/science.8493529 8493529

[B33] Lee D. H. Tamura A. Arisaka Y. Seo J.-H. Yui N. (2019). Mechanically Reinforced Gelatin Hydrogels by Introducing Slidable Supramolecular Cross-Linkers. Polymers 11, 1787. 10.3390/polym11111787 PMC6918157 31683825

[B34] Ma L. Yu Y. Liu H. Sun W. Lin Z. Liu C. (2021). Berberine-releasing Electrospun Scaffold Induces Osteogenic Differentiation of DPSCs and Accelerates Bone Repair. Scientific Rep. 11, 1–12. 10.1038/s41598-020-79734-9 PMC7806735 33441759

[B35] Miller J. S. Stevens K. R. Yang M. T. Baker B. M. Nguyen D.-H. T. Cohen D. M. (2012). Rapid Casting of Patterned Vascular Networks for Perfusable Engineered Three-Dimensional Tissues. Nat. Mater 11, 768–774. 10.1038/nmat3357 22751181 PMC3586565

[B36] Morrow C. M. Mukherjee A. Traore M. A. Leaman E. J. Kim A. Smith E. M. (2019). Integrating Nanofibers with Biochemical Gradients to Investigate Physiologically-Relevant Fibroblast Chemotaxis. Lab. Chip 19, 3641–3651. 10.1039/c9lc00602h 31560021

[B37] Mou L. Jiang X. (2017). Materials for Microfluidic Immunoassays: A Review. Adv. Healthc. Mater. 6, 1601403. 10.1002/adhm.201601403 28322517

[B38] Murphy K. C. Leach J. K. (2012). A Reproducible, High Throughput Method for Fabricating Fibrin Gels. BMC Res. Notes 5, 423–424. 10.1186/1756-0500-5-423 22873708 PMC3492004

[B39] Papaefthymiou G. C. (2013). “Microfluidic-Based Polymer Scaffold Design for Tissue Engineering,” in Nanobiomaterials (Boca Raton, FL: CRC Press), 359–378.

[B40] Pimentel C. R. Ko S. K. Caviglia C. Wolff A. Emnéus J. Keller S. S. (2018). Three-dimensional Fabrication of Thick and Densely Populated Soft Constructs with Complex and Actively Perfused Channel Network. Acta Biomater. 65, 174–184. 10.1016/j.actbio.2017.10.047 29102798

[B41] Pomerantseva I. Krebs N. Hart A. Neville C. M. Huang A. Y. Sundback C. A. (2009).Degradation Behavior of Poly(glycerol Sebacate). J. Biomed. Mater. Res., 91 (4), 1038–1047. 10.1002/jbm.a.32327 19107788

[B42] Raman P. S. Paul C. D. Stroka K. M. Konstantopoulos K. (2013). Probing Cell Traction Forces in Confined Microenvironments. Lab. Chip 13, 4599–4607. 10.1039/c3lc50802a 24100608 PMC5409513

[B43] Ren K. Zhou J. Wu H. (2013). Materials for Microfluidic Chip Fabrication. Acc. Chem. Res. 46, 2396–2406. 10.1021/ar300314s 24245999

[B44] Rinawati M. Triastuti J. Pursetyo K. (2018). “Characterization of Elasticity and Hydration of Composite Hydrogel Based on Collagen-iota Carrageenan as a Corneal Tissue Engineering,” in IOP Conference Series: Earth and Environmental Science, Banda Aceh, Indonesia, 26–27 September 2018 (Bristol, England: IOP Publishing), 012042.

[B45] Saito T. Tabata Y. (2012). Preparation of Gelatin Hydrogels Incorporating Low-Molecular-Weight Heparin for Anti-fibrotic Therapy. Acta Biomater. 8, 646–652. 10.1016/j.actbio.2011.10.025 22079782

[B46] Scotti C. Tonnarelli B. Papadimitropoulos A. Scherberich A. Schaeren S. Schauerte A. (2010). Recapitulation of Endochondral Bone Formation Using Human Adult Mesenchymal Stem Cells as a Paradigm for Developmental Engineering. Proc. Natl. Acad. Sci. 107, 7251–7256. 10.1073/pnas.1000302107 20406908 PMC2867676

[B47] Shan D. Gerhard E. Zhang C. Tierney J. W. Xie D. Liu Z. (2018). Polymeric Biomaterials for Biophotonic Applications. Bioactive Mater. 3, 434–445. 10.1016/j.bioactmat.2018.07.001 PMC6086320 30151431

[B48] Shen C. Li Y. Wang Y. Meng Q. (2019). Non-swelling Hydrogel-Based Microfluidic Chips. Lab. Chip 19, 3962–3973. 10.1039/c9lc00564a 31656966

[B49] Shin H. Olsen B. D. Khademhosseini A. (2012). The Mechanical Properties and Cytotoxicity of Cell-Laden Double-Network Hydrogels Based on Photocrosslinkable Gelatin and Gellan Gum Biomacromolecules. Biomaterials 33, 3143–3152. 10.1016/j.biomaterials.2011.12.050 22265786 PMC3282165

[B50] Shkand T. V. Chizh M. O. Sleta I. V. Sandomirsky B. P. Tatarets A. L. Patsenker L. D. (2016). Assessment of Alginate Hydrogel Degradation in Biological Tissue Using Viscosity-Sensitive Fluorescent Dyes. Methods Appl. Fluoresc. 4, 044002. 10.1088/2050-6120/4/4/044002 28192295

[B51] Skornia S. L. Bledsoe J. G. Kelso B. Kuntz Willitz R. (2007). Mechanical Properties of Layered Poly (Ethylene Glycol) Gels. J. Appl. Biomater. Biomech. 5, 176–183. 10.1177/228080000700500306 20799187

[B52] Sukpaita T. Chirachanchai S. Suwattanachai P. Everts V. Pimkhaokham A. Ampornaramveth R. S. (2019). *In Vivo* bone Regeneration Induced by a Scaffold of Chitosan/dicarboxylic Acid Seeded with Human Periodontal Ligament Cells. Int. J. Mol. Sci. 20, 4883. 10.3390/ijms20194883 PMC6801435 31581495

[B53] Thomas S. Chandra A. K. Visakh P. (2012). Advances in Elastomers II: Composites and Nanocomposites. Berlin/Heidelberg, Germany: Springer.

[B54] Tong A. Pham Q. L. Abatemarco P. Mathew A. Gupta D. Iyer S. (2021). Review of Low-Cost 3D Bioprinters: State of the Market and Observed Future Trends. SLAS Technol., 24726303211020297, 10.1177/24726303211020297 34137286

[B55] Tong A. Pham Q. L. Shah V. Naik A. Abatemarco P. Voronov R. (2020). Automated Addressable Microfluidic Device for Minimally Disruptive Manipulation of Cells and Fluids within Living Cultures. ACS Biomater. Sci. Eng. 6, 1809–1820. 10.1021/acsbiomaterials.9b01969 33455370

[B56] Tran R. T. Thevenot P. Gyawali D. Chiao J.-C. Tang L. Yang J. (2010). Synthesis and Characterization of a Biodegradable Elastomer Featuring a Dual Crosslinking Mechanism. Soft matter 6, 2449–2461. 10.1039/c001605e 22162975 PMC3233194

[B57] Trujillo S. Gonzalez-Garcia C. Rico P. Reid A. Windmill J. Dalby M. J. (2019). Engineered Full-Length Fibronectin-Based Hydrogels Sequester and Present Growth Factors to Promote Regenerative Responses *in vitro* and *in vivo* . bioRxiv, 687244. 10.1101/687244

[B58] Underwood S. Afoke A. Brown R. A. Macleod A. J. Shamlou P. A. Dunnill P. (2001). Wet Extrusion of Fibronectin-Fibrinogen Cables for Application in Tissue Engineering. Biotechnol. Bioeng. 73, 295–305. 10.1002/bit.1062 11283912

[B59] Ürek H. Özdemir E. Coramik M. (2021). Using Tracker to Find the Minimum Angle of Deviation and the Refractive index of a Prism. Phys. Edu. 56, 035016. 10.1088/1361-6552/abe3cb

[B60] Vogt L. Ruther F. Salehi S. Boccaccini A. R. (2021). Poly(Glycerol Sebacate) in Biomedical Applications-A Review of the Recent Literature. Adv. Healthc. Mater. 10, 2002026. 10.1002/adhm.202002026 PMC11468981 33733604

[B61] Wang J. Bettinger C. J. Langer R. S. Borenstein J. T. (2010). Biodegradable Microfluidic Scaffolds for Tissue Engineering from Amino Alcohol-Based Poly(ester Amide) Elastomers. Organogenesis 6, 212–216. 10.4161/org.6.4.12909 21220957 PMC3055646

[B62] Wu P.-H. Giri A. Sun S. X. Wirtz D. (2014). Three-dimensional Cell Migration Does Not Follow a Random Walk. Proc. Natl. Acad. Sci. 111, 3949–3954. 10.1073/pnas.1318967111 24594603 PMC3964056

[B63] Wu Y. Xiang Y. Fang J. Li X. Lin Z. Dai G. (2019). The Influence of the Stiffness of GelMA Substrate on the Outgrowth of PC12 Cells. Biosci. Rep. 39. 10.1042/BSR20181748 PMC6340955 30606743

[B64] Xie R. Zheng W. Guan L. Ai Y. Liang Q. (2020). Engineering of Hydrogel Materials with Perfusable Microchannels for Building Vascularized Tissues. Small 16, 1902838. 10.1002/smll.201902838 31559675

[B65] Ye K. Kaplan D. L. Bao G. Bettinger C. Forgacs G. Dong C. (2018). Advanced Cell and Tissue Biomanufacturing. ACS Biomater. Sci. Eng. 4, 2292–2307. 10.1021/acsbiomaterials.8b00650 33435095

[B66] Yildirimer L. Thanh N. T. K. Seifalian A. M. (2012). Skin Regeneration Scaffolds: a Multimodal Bottom-Up Approach. Trends Biotechnology 30, 638–648. 10.1016/j.tibtech.2012.08.004 22981509

[B67] Zhang B. Lai B. F. L. Xie R. Davenport Huyer L. Montgomery M. Radisic M. (2018). Microfabrication of AngioChip, a Biodegradable Polymer Scaffold with Microfluidic Vasculature. Nat. Protoc. 13, 1793–1813. 10.1038/s41596-018-0015-8 30072724

[B68] Zhang Y. S. Davoudi F. Walch P. Manbachi A. Luo X. Dell'erba V. (2016). Bioprinted Thrombosis-On-A-Chip. Lab. Chip 16, 4097–4105. 10.1039/c6lc00380j 27722710 PMC5072176

[B69] Zhao S. Chen Y. Partlow B. P. Golding A. S. Tseng P. Coburn J. (2016). Bio-functionalized Silk Hydrogel Microfluidic Systems. Biomaterials 93, 60–70. 10.1016/j.biomaterials.2016.03.041 27077566

[B70] Zhu M. Wang Y. Ferracci G. Zheng J. Cho N. J. Lee B. H. (2019). Gelatin Methacryloyl and its Hydrogels with an Exceptional Degree of Controllability and Batch-To-Batch Consistency. Sci. Rep. 9, 6863. 10.1038/s41598-019-42186-x 31053756 PMC6499775

